# Prolonged versus brief balloon inflation during arterial angioplasty for de novo atherosclerotic disease: protocol for a systematic review

**DOI:** 10.1186/s13643-019-0955-2

**Published:** 2019-02-05

**Authors:** Mark Rockley, Prasad Jetty, George Wells, Kathleen Rockley, Dean Fergusson

**Affiliations:** 1 Division of Vascular and Endovascular Surgery, Department of Surgery, University of Ottawa, The Ottawa Hospital - Civic Campus, Ottawa, K1Y4E9 Canada; 2 Cardiovascular Research Methods Centre, University of Ottawa Heart Institute, Ottawa, K1Y4W7 Canada; 3 Clinical Epidemiology Program, Ottawa Hospital Research Institute, Ottawa, K1H8L6 Canada

**Keywords:** Angioplasty, Duration, Prolonged, Restenosis, Arterial patency

## Abstract

**Background:**

Angioplasty is a fundamental treatment for atherosclerotic disease in the cardiac, cerebrovascular, and peripheral vascular beds. However, the optimal duration of balloon inflation has not been identified. Our study will investigate whether prolonged angioplasty balloon inflation of at least 1 min duration, when compared with brief inflation, affects residual stenosis after arterial angioplasty.

**Methods:**

In compliance with PRISMA, two independent reviewers will conduct a systematic review of EMBASE, MEDLINE, CENTRAL, trial registries, grey literature, and ancestry and citation search. Data abstraction, quantitative, and quantitative meta-analysis will be performed according to pre-specified criteria. The primary outcome is residual stenosis immediately after initial angioplasty; however, secondary outcomes will include multiple short and long term pre-specific clinical and radiographic outcomes. Risk of bias, subgroup analyses, and sensitivity analyses are planned.

**Discussion:**

Despite the ubiquitous use of angioplasty in atherosclerotic disease and multiple trials investigating the ideal balloon inflation duration, there are no systematic reviews evaluating prolonged angioplasty balloon inflation. Currently synthesized evidence is insufficient to confidently direct clinical decision-making, and the current variation in operator preference of balloon angioplasty duration suggests ongoing clinical equipoise. Given the known availability of current primary evidence, our study intends to synthesize the evidence and guide future clinical decision making and investigation.

**Systematic review registration:**

PROSPERO CRD42018092702

**Electronic supplementary material:**

The online version of this article (10.1186/s13643-019-0955-2) contains supplementary material, which is available to authorized users.

## Background

### Rationale

Atherosclerotic vascular disease is a chronic disease that causes blockages of arteries throughout the body. Conditions resulting from atherosclerotic disease are the most common cause of death in North America. The location of the vascular blockage dictates the disease; blockages in coronary arteries may lead to heart attacks, blockages in cerebral arteries may lead to strokes, and blockages in peripheral arteries may lead to gangrene. The prevalence of atherosclerotic peripheral vascular disease in the North American general population over 50 years of age is estimated at 17.4% [1] and is rising in association with the increasing prevalence of diabetes. While bypass surgery is reserved for select patients with severe forms of atherosclerotic disease, the minimally invasive options of angioplasty are the initial treatment of choice for most patients. Angioplasty is the foundational treatment of endovascular therapy, which may be augmented by treatments such as stenting or atherectomy. Unfortunately, the 2-year patency of plain balloon angioplasty for peripheral vascular disease has been poor, reported between 50 and 80%, depending on lesion location and characteristics [2].

Various techniques have been proposed to minimize restenosis following angioplasty; however, many have not been thoroughly evaluated in randomized trials. One such technique is prolonged angioplasty balloon inflation time, which is theorized to reduce post-interventional dissection and induce smooth muscle dysfunction, thereby reducing vasospasm and resulting stenosis. While animal studies have not revealed short-term mechanical advantage for prolonged balloon inflation [3], smooth muscle dysfunction has been observed [4]. Both of these studies compared brief balloon inflation times less than 60 s with inflation times greater than 60 s. Alternatively, prolonged balloon inflation may incur risk to the patient by simply mask flow-limiting dissections, which could have been identified if only transient balloon inflation was used. Identification of these dissections is relevant as they are at high risk of causing target lesion occlusion and necessitate stent placement.

Conversely, in addition to being a therapeutic treatment for atherosclerotic disease, balloon angioplasty is known to cause vascular damage to previously healthy arteries in animal models after an inflation time of 60 s [5–7]. These articles present several proposed mechanisms for arterial injury during angioplasty; however, they all ultimately refer to intimal hyperplasia which occurs within hours to days after the initial angioplasty, due to inflammatory response following endothelial injury caused by the balloon inflation. This contradiction raises concern that although angioplasty may result in esthetic immediate results, there is a fundamental limitation to preserving long-term results after angioplasty. In particular, prolonged angioplasty balloon inflation may result in superior immediate results, but may also cause more profound vascular injury. The potential risks to patients of long-term vascular injury due to prolonged angioplasty is currently unknown.

When angioplasty was first introduced in the last twentieth century, early randomized trials revealed immediate benefit of prolonged balloon inflation, although the sustainability of the patency benefit is conflicting [8–10]. However, the immediate benefit of prolonged angioplasty has not been consistently reported in randomized trials [11]. Initial studies describing the methods of arterial angioplasty in peripheral arteries use 1 min duration inflations without justification for the inflation duration [12]. Despite the ubiquitous use of angioplasty in atherosclerotic disease and ongoing primary investigation, there are no systematic reviews evaluating prolonged angioplasty balloon inflation. Currently synthesized evidence is insufficient to confidently direct clinical decision-making, and the current variation in operator preference of balloon angioplasty duration suggests ongoing clinical equipoise.

### Objectives

The objective of this systematic review is to evaluate the risks and benefits of prolonged angioplasty balloon inflation duration in the diverse patients with atherosclerotic disease.

Specifically, our primary objective will be to determine if in patients receiving elective arterial angioplasty for stenotic or occlusive atherosclerotic disease, does prolonged balloon inflation of greater than 1 min duration, when compared with brief balloon inflation 1 min or less, improve residual stenosis in the immediate post-procedure angiogram.

Secondary questions that will be addressed by this review will include:

1. Is the effect of prolonged balloon inflation modulated by the location of atherosclerotic disease: coronary, cerebrovascular, and peripheral?
2. Does prolonged balloon inflation, when compared with brief balloon inflation, affect immediate adverse radiographic events such as major vascular dissection?
3. Does prolonged balloon inflation, when compared with brief balloon inflation, affect immediate clinically apparent adverse events secondary to vessel territory ischemia, such as heart attack or stroke?
4. Does prolonged balloon inflation, when compared with brief balloon inflation, affect long term radiographic vessel patency?
5. Does prolonged balloon inflation, when compared with brief balloon inflation, affect long term resolution of clinical ischemic symptoms?

## Methods

### Eligibility criteria

#### Study designs

We will include randomized controlled trial (RCT), including cluster RCT, non-randomized controlled trials, cluster trials, interrupted time series studies, controlled before-after studies (CBA), and prospective or retrospective cohort studies. Case series, nested case-control, cross-sectional studies, and case reports will be excluded.

#### Participants

We will include studies examining human adults (age 18 or older) who received angioplasty for atherosclerotic stenotic or obstructive vascular disease. Subgroup analysis examining clinical symptoms will exclude all trials that do not reference ischemia-based symptoms and only report radiographic results. Of note, when multiple arterial lesions are intervened upon within the same patient, each lesion will be counted as a separate “participant.”

#### Intervention and comparators

We will examine studies investigating elective arterial angioplasty. The angioplasty must be the primary purpose of the intervention and not be performed concurrently with a hybrid open vascular procedure on an in-line flow artery. The intervention may be performed on any arterial structure, including coronary, extracranial, intracerebral, or extremity arteries. The balloons may be drug-coated or lined with cutting ribs. However, we will exclude studies which employed adjunctive endovascular procedures prior to measurement of the outcome of interest, including but not limited to stent placement, orbital atherectomy, laser atherectomy, rotational atherectomy, or directional atherectomy. We will exclude venous angioplasty, arteriovenous fistula angioplasty, and studies examining emergency settings. We will only include studies examining de novo stenosis and therefore exclude studies examining angioplasty of restenosis, as the histopathology and outcomes related to restenosis are vastly different than de novo atherosclerosis [13].

The intervention of interest is at least one single prolonged angioplasty balloon inflation greater than 1 min duration, and the control will be brief balloon inflation, with no single balloon inflation duration greater than 1 min.

#### Outcomes

The primary outcome of interest is residual stenosis immediately following angioplasty, as determined by post-inflation angiogram. The definition threshold for residual stenosis must be defined and is generally between 20 and 50% stenosis.

Secondary endpoints are categorized into immediate and long-term outcomes. Immediately, we will collect reported information on radiographic adverse events; this excludes residual stenosis and will be defined as a vascular anatomic abnormality that did not exist prior to balloon inflation. This includes arterial dissection, vessel perforation, and acute occlusion where there was a degree of patency prior to intervention. In addition to radiographic adverse events, we will collect newly developed clinically significant adverse events noted during the day of intervention, which broadly encompasses ischemic symptoms secondary to the territory of vascular intervention, for instance, a myocardial infarction following coronary artery angioplasty, stroke following cerebral vessel angioplasty, or acute limb ischemia following peripheral arterial angioplasty.

Long-term secondary endpoints will also be differentiated into radiographic or clinical outcomes. We will collect data from any study reporting radiographic vessel patency by any validated modality, when performed on the day following the initial intervention: interventional angiogram, CT-angiogram, MRI-angiogram, and duplex ultrasound will be considered. Clinically, symptoms indicating long term resolution of ischemia will include angina pectoralis, cerebral hypoperfusion syndromes, claudication, or critical limb ischemia.

#### Timing

Studies will be selected for inclusion if they report immediate post-angioplasty angiographic results, performed on the same day as the procedure. Long-term endpoint data will also be collected, as described above, if available.

#### Setting

There are no restrictions regarding the setting of the study.

#### Language

We will include English and French studies, with a list of potentially relevant translated titles in other languages included in an Additional file 1.

### Information sources

A literature search strategy using medical subject headings and text words has been developed. We will search MEDLINE (OVID interface), EMBASE (OVID interface), and the Cochrane Central Register of Controlled Trials (Wiley interface).

To ensure capture of all relevant trials, all selected studies will also undergo ancestry search, in addition to citation search using SCOPUS. OpenGrey will be interrogated for unpublished relevant literature.

### Search strategy

Both qualitative and quantitative studies will be collected. All searches will be limited by date of publication (January 1977–February 2018). The initial year of 1977 has been chosen as the first in-human use of angioplasty was performed that year. No language limit will be placed on the search; however, only English or French studies will be included in the analysis, with titles of potentially relevant studies in alternate languages included in the Additional file 1. The search strategy and syntax will be guided by a Health Sciences librarian with systematic review experience. After the MEDLINE search syntax has been finalized, it will be adapted to accommodate the remaining database searches. Please see Additional file 2 for a complete search syntax used for the MEDLINE search. Of note, the PROSPERO database has been searched, and no ongoing or recently completed systematic review on this topic has been performed.

### Study records

#### Data management

Literature search results will be aggregated in EndNote, including where duplicate articles will be removed. The results will then be uploaded to the Distiller SR software, which will facilitate collaboration among all reviewers.

The two screening authors will independently screen titles and abstracts resulting from the combined search of all selected databases. The full text of an article will be obtained for any articles that appear to meet eligibility criteria, at which point the full text will be screened and confirmation of article inclusion will be made. Any reasons for exclusion following full text screening will be explicitly documented and listed in an Additional file 1.

Once both reviewers have created a complete list of eligible articles, the lists will be compared. Discrepancies in article selection will be addressed with discussion with a third-party author experienced in systematic review conduct. No authors will be blinded to journal titles, study authors, or study location of origin.

#### Data collection process

A standardized form created in Distiller SR will be used as the data collection method. Both reviewers will have a separate form for each article, which will be compared for consistency after data collection has completed. Any discrepancy will be addressed with discussion with a third-party author experienced in systematic review conduct. Study authors will not be contacted to resolve unclear or inadequate reporting of data.

### Data items

Generic article data collected will include year of publication, trial design methodology, trial size (both in number of patients and number of lesions), duration of follow-up, financial support sources and involvement, and publication status.

Patient-specific data will include average age, gender, symptom status, and the anatomic location of arterial lesions (classified as coronary, cerebrovascular, peripheral, and other).

Intervention-specific data will include the trial protocol for angioplasty balloon inflation duration, the total number of repeated inflations, target balloon inflation pressure, use of adjunctive endovascular therapy following angioplasty, and the total number of lesions intervened upon in each intervention arm. We will also document the type of angioplasty device used; specifically, we will record any use of drug-eluting balloons, cutting rib balloons, and perfusion catheters.

Outcome-specific data will include the blinding status of outcome adjudication, the definition of restenosis, definition of acute or chronic radiologic or symptomatic adverse events, and any long-term vessel patency or symptom status outcomes.

### Outcomes and prioritization

#### Primary outcome

The primary outcome will be the number of lesions who do not suffer from residual stenosis immediately following angioplasty balloon inflation, with a threshold as defined specifically by the individual trials but not exceeding the limits of 20–50% residual stenosis. We will record whether the treating physician or a blinded adjudicator determined the end-point.

### Secondary outcomes

1. Immediate outcomes
  - Radiographic
    i. Defined as a vascular anatomic abnormality that did not exist prior to balloon inflation. We will record whether the treating physician or a blinded adjudicator determined the end-point. These will be recorded as dichotomous outcomes.
      1. Vessel dissection
      2. Vessel perforation
      3. Distal embolization
      4. Composite endpoint, which may include dissection, perforation, or severe residual stenosis
  - Clinical
    i. Defined as newly developed ischemic symptoms, temporally related to intervention, and anatomically related to ischemia of the end-organ perfused by the artery receiving angioplasty. These will be recorded as dichotomous outcomes.
      1. Myocardial infarction or angina pectoralis
        - Clinical or biochemical diagnostic confirmation
      2. Stroke or transient ischemic attack
        - Clinical diagnosis, with or without radiographic confirmation
      3. Acute limb ischemia
        - Clinical diagnosis, with or without radiographic confirmation
2. Long-term outcomes
  - Radiographic
    i. Defined as any record of radiographic vessel patency by any validated modality, when performed on the day following the initial intervention. The length of follow-up will be noted, and multiple records kept if follow-up images were obtained at multiple time point following the intervention. We will record whether the treating physician or a blinded adjudicator determined the end-point. These will be recorded as dichotomous outcomes.
      1. Interventional angiogram
      2. CT angiogram
      3. MR angiogram
      4. Duplex ultrasound
  - Clinical
    i. Defined as the recurrence or development of chronic ischemic symptoms related to the end-organ perfused by the vessel intervened upon by angioplasty. These will be recorded as dichotomous outcomes.
      1. Angina pectoralis
      2. Cerebral hypoperfusion syndrome
      3. Claudication
      4. Critical limb ischemia.

### Risk of bias of individual studies

To assess individual studies for potential risk of bias, we will collect information guided by the Cochrane Collaboration tool for assessing risk of bias. In summary, this includes sequence generation, allocation concealment, blinding, incomplete outcome data, and selective outcome reporting. For each category, each study will be determined to be at either low or high risk. Alternately, if the report includes insufficient information to determine the level of risk, the category will be labeled as unclear. Determination of the level of bias will be made by the two reviewers independently and compared following complete assessment of all studies. Any discrepancy will be addressed with discussion with a third-party author experienced in systematic review conduct. The resulting risk of bias for each study, in each category, will be graphically represented by RevMan software.

### Data synthesis

#### Quantitative synthesis

Following data collection, if the studies are homogenous in terms of design, subjects, interventions, and outcomes, we will conduct a meta-analysis with random effects model. All outcomes that may undergo meta-analysis will be dichotomous in nature. Measurement effects will be determined by using risk ratio with 95% confidence intervals. As mentioned, the proposed unit of analysis will be arterial lesions; one subject may lend multiple units of analyses if they have multiple lesions undergoing angioplasty. Clustering of data for purposes of meta-analyses will not be considered due to variable reporting patterns, resulting in statistical limitations. However, for cluster randomized trials, we will account for the interclass correlation coefficient to appropriately modify results, as guided by the Cochrane Handbook for Systematic Reviews of Interventions.

Due to the anticipated heterogeneous nature of clinical symptomatic status presentation and reporting, in particular given the broad anatomic inclusion of this study, clinical symptom outcomes will not be assessed using quantitative meta-analysis.

#### Issues relating to data quality

In cases of unclear or inadequate data reporting, the authors will not be contacted for further data, and the data will be absent from meta-analysis. For trials that did not report on an intention-to-treat basis, or otherwise are at unique risk for missing or high risk of bias, sensitivity analysis will be used to assess the effect of inclusion of these trials. Trials will not be excluded due to the number of participants, however, will be assessed with sensitivity analysis if the size of trials raises heterogeneity concerns.

To assess for study heterogeneity, we will first subjectively assess variability in patient and study baseline factors, intervention type, and outcome assessments. Statistical heterogeneity will be tested using the *I*^2^ statistic, with a threshold of 40–60% possibly representing heterogeneity and greater than 60% likely representing heterogeneity. In cases of likely heterogeneity, we will attempt to identify the source of heterogeneity through the use of pre-defined subgroup or post hoc sensitivity analysis.

#### Quantitative data synthesis

All quantitative data syntheses will be performed on RevMan software as guided by the Cochrane Handbook for Systematic Reviews of Interventions. If acceptable levels of homogeneity are observed, cumulative effect estimates will be calculated with the Mantel-Haenszel method using fixed effects model. Alternately, a qualitative synthesis will be performed.

#### A priori subgroup analyses

Planned subgroup analyses include:

- Anatomic location of arterial intervention (coronary, cerebrovascular, peripheral, and other)
- Number of balloon inflations (single inflation versus repeated inflations)
- Era of study (prior to year 2000 and following year 2000)
- Angioplasty device (plain balloon, drug-coated balloon, cutting balloon, perfusion catheter)

#### Qualitative synthesis

All reported outcomes will be synthesized and reported in a qualitative manner. Furthermore, clinical outcomes will only be synthesized in a qualitative manner, as the expected heterogeneity in clinical situations and reporting will preclude quantitative analysis.

#### Meta-bias

In addition to individual study assessment of risk of bias, all studies will be evaluated for indications of meta-bias. We will search for preceding published or registered protocols prior to study publication and evaluate for selective outcome reporting. Potential for reporting bias will be assessed by a funnel plot.

### Confidence in cumulative estimate

The quality of all outcomes will be judged subjectively as a consensus among study authors, using the standardized Grading of Recommendations Assessment, Development and Evaluation methodology.

## Discussion

This study intends to focus on a procedural technique for a systemic disease and encompasses a broad scope of specialties and anatomic regions. This breadth is intentional and intended to account for the relatively low anticipated body of evidence on this topic. However, the breadth of the study also introduces limitations during the conduct and interpretation of this review. While a meta-regression to determine the relative impact of angioplasty inflation duration on restenosis could theoretically demonstrate an ideal inflation duration, this will not be feasible due to the anticipated breadth of disease and reporting variability. Therefore, the primary conclusion of this study will determine whether balloon inflation of greater than 60 s is favorable; the specific ideal duration will not be determined in the context of these limitations. Furthermore, case-specific characteristics that are known to affect angioplasty success, such as other systemic diseases and lesion characteristics, will not be feasible to introduce into the analysis. Therefore, this study relies on the integrity of study allocation procedures to ensure the treatment arms are comparable. Despite these limitations, the potential results of this study remain insightful and necessary in light of the ubiquity of angioplasty and variations in practice. Adherence to the PRISMA-P guidelines (see Additional file 2) will assist in performing a transparent and thorough investigation.

## Additional files

Additional file 1: Proposed search syntax for MEDLINE, using OVID interface. (LOG 100 bytes). (DOCX 24 kb)

Additional file 2: PRISMA-P 2015 Checklist. (DOCX 33 kb)

## Abbreviations

CBA: Controlled before-after trial
RCT: Randomized control trial
