# Supplementary material for: Prolonged versus brief balloon inflation during arterial angioplasty for de novo atherosclerotic disease: protocol for a systematic review
Source: Syst Rev. 2019 Feb 5;8:45. doi: 10.1186/s13643-019-0955-2 (PMC6362580; doi:10.1186/s13643-019-0955-2)
Supplement: Supplementary file 1 — Proposed search syntax for MEDLINE, using OVID interface. (LOG 100 bytes). (DOCX 24 kb) [file 13643_2019_955_MOESM1_ESM.docx]

**Additiona file 1: Appendix**

**Angioplasty**

1. ANGIOPLASTY, BALLOON/ or ANGIOPLASTY, BALLOON, CORONARY/

2. (balloon* adj2 angioplast*).tw.

3. (transluminal adj3 arteri* adj3 dilation*).tw.

4. (balloon* adj2 inflat*).tw.

**Residual Stenosis**

5. Constriction, Pathologic/

6. Vascular Patency/

7. (residual adj2 stenos*).tw.

8. (angiogra* adj2 result*).tw.

9. (unstable adj2 lesion*).tw.

10. (morpholog* adj2 result*).tw.

11. (radio* adj2 result*).tw.

**Balloon Inflation Time**

12. Time Factors/

13. prolonged.tw.

14. duration.tw.

15. brief.tw.

16. (inflation adj2 time*).tw.

17. patency.tw.

**Combination of Search Concepts**

18. 1 or 2 or 3 or 4

19. 5 or 6 or 7 or 8 or 9 or 10 or 11 or 17

20. 12 or 13 or 14 or 15 or 16

21. 18 and 19 and 20

**Search Limits**

22. limit 21 to "all adult (19 plus years)"

23. limit 22 to yr="1977 - 2018"

**Additional file 1: Appendix.** Proposed search syntax for MEDLINE, using OVID interface
